# The Lao Experience in Deploying Influenza A(H1N1)pdm09 Vaccine: Lessons Made Relevant in Preparing for Present Day Pandemic Threats

**DOI:** 10.1371/journal.pone.0121717

**Published:** 2015-04-29

**Authors:** Anonh Xeuatvongsa, Sara Mirza, Christian Winter, Keith Feldon, Phengta Vongphrachanh, Darouny Phonekeo, Justin Denny, Viengphone Khanthamaly, Bounheuang Kounnavong, Doualy Lylianou, Sisouphane Phousavath, Sisouveth Norasingh, Nao Boutta, Sonja Olsen, Joseph Bresee, Ann Moen, Andrew Corwin

**Affiliations:** 1 National Immunization Program, Ministry of Health, Vientiane, Lao People’s Democratic Republic (Lao PDR); 2 Influenza Division, United States Centers for Disease Control and Prevention, Atlanta, Georgia, United States of America; 3 Emerging Diseases Surveillance and Response Unit, World Health Organization-Western Pacific Region, Country Office, Vientiane, Lao PDR; 4 Expanded Program on Immunization, World Health Organization, Country Office, Vientiane, Lao PDR; 5 National Center for Laboratory and Epidemiology, Ministry of Health, Vientiane, Lao PDR; 6 United States Centers for Disease Control and Prevention, Country Office, Vientiane, Lao PDR; 7 Field Epidemiology Training Program, National Center for Laboratory and Epidemiology, Ministry of Health, Vientiane, Lao PDR; 8 Cabinet, Ministry of Health, Vientiane, Lao PDR; University of Edinburgh, UNITED KINGDOM

## Abstract

The Lao PDR, as did most countries of the Mekong Region, embarked on a pandemic vaccine initiative to counter the threat posed by influenza A(H1N1)pdm09. Overall, estimated vaccine coverage of the Lao population was 14%, with uptake in targeted health care workers and pregnant women 99% and 41%, respectively. Adverse Events Following Immunization accounted for only 6% of survey driven, reported vaccination experiences, with no severe consequences or deaths. Public acceptability of the vaccine campaign was high (98%). Challenges to vaccine deployment included: 1) no previous experience in fielding a seasonal influenza vaccine, 2) safety and efficacy concerns, and 3) late arrival of vaccine 10 months into the pandemic. The Lao success in surmounting these hurdles was in large measure attributed to the oversight assigned the National Immunization Program, and national sensitivities in responding to the avian influenza A(H5N1) crisis in the years leading up to the pandemic. The Lao “lessons learned” from pandemic vaccine deployment are made even more relevant four years on, given the many avian influenza strains circulating in the region, all with pandemic potential.

## Background

**Fig 1:**
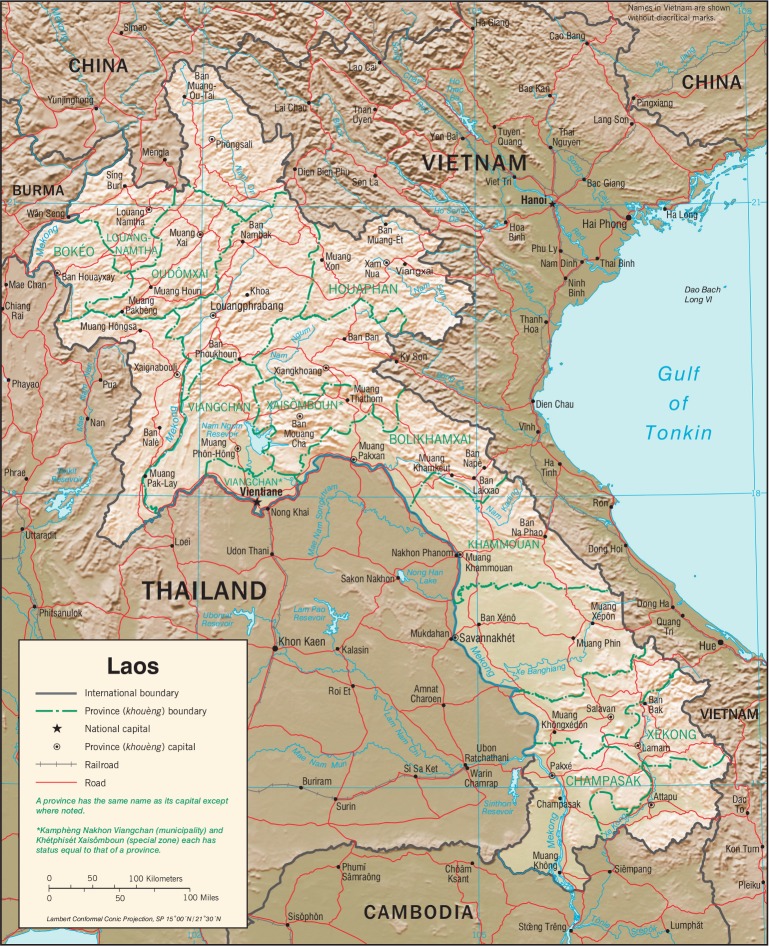
The Mekong Region: Lao PDR Sharing Border with Cambodia, China, Myanmar, Thailand and Vietnam. Source: https://www.cia.gov/library/publications/cia-maps-publications/Laos.html.

In April 2009, the first cases of a new swine-origin influenza virus A (H1N1) pdm09 were identified in the United States [1]. The virus quickly spread globally, and the World Health Organization (WHO) declared a pandemic on June 11, 2009 [2, 3]. As part of the global public health response, WHO launched the Pandemic Influenza A(H1N1) Vaccine Deployment Initiative (PIVDI) to promote and coordinate donation activities across governments, public health partners and manufacturers. Ninety-five countries worldwide lacking local vaccine production and resources to fund vaccines purchases were found eligible for vaccine donations, including four countries in the Mekong Region: Cambodia, Myanmar, Vietnam and Lao PDR [4] (Fig 1).

Introduction of influenza A(H1N1)pdm09 into the Mekong region, galvanized health authorities to embark on a vaccine intervention strategy. Three countries supported by the WHO PIVDI initiated orders for the influenza A(H1N1)pdm09 monovalent vaccine: Cambodia ordered 2,712,000 doses, Myanmar 972,000 doses and Lao PDR 1,000,000 doses; providing for population coverage estimates of 2%, 18% and 14% respectively [5]. Vietnam while eligible chose not to participate through PIVDI, and Thailand purchased 2 million doses, as it was not eligible for PIVDI.

Except for Thailand, none of the other 4 countries in the region had public programs for seasonal influenza immunization. Even so, the region was sensitized to avian influenza A(H5N1) virus and its pandemic potential. Significant investments in avian and seasonal influenza laboratory diagnostics and surveillance, spurred by emergence of avian influenza A(H5N1) in 2004, yielded new detection and response capabilities that generated evidence driven findings as to local influenza seasonality, circulating subtypes, and the relative contribution of influenza to respiratory illness These capabilities enabled early recognition of the introduction of pandemic influenza into the Mekong area.

Current pandemic and novel influenza threats facing the region include the emergence of avian influenza A(H7N9) in China, the persistent threat of avian influenza A(H5N1) (reflecting in ongoing circulation in Cambodia), the recent detection of the first human case and fatality attributed to A(H5N6) in China (February 2014) and the first and second poultry outbreaks in Lao PDR (March and July 2014), and the ongoing potential for re-assortment through mixing of seasonal and avian influenza virus into more lethal transmissible sub-strains (Table 1). In this paper, we review the Lao experience with (2009/10) pandemic influenza vaccine, and find valuable “lessons learned”, unique to Lao PDR and shared by the region that can be applied in countering current and future pandemic threats. Moreover, findings reflect the importance of vaccine considerations as an integral part of pandemic preparedness planning.

**Table 1:** Pandemic Avian Influenza Threats to the Mekong Region, 2013–2014.

	Human cases						Animal outbreaks					
	H5N1		H5N6		H7N9		H5N1		H5N6		H7N9	
	2013	2014	2013	2014	2013	2014	2013	2014	2013	2014	2013	2014
**Cambodia**	26^a^	9^a^					7^c^	5^c^				
**China**	2^a^	2^a^		2^b^	156^b^	309^b^	4^c^	7^c^		5^c^		
**Lao PDR**										2^c^		
**Myanmar**												
**Thailand**												
**Vietnam**	2^a^	2^a^					6^c^	38^c^		5^c^		
**Total:**	**30**	**13**		**2**	**156**	**309**	**17**	**50**		**12**		

Source:^a^WHO/GIP, data in HQ as of 6 January 2015 http://www.who.int/influenza/human_animal_interface/EN_GIP_20150106CumulativeNumberH5N1cases.pdf. Accessed 29 January 2015

^b^ http://novel-infectious-diseases.blogspot.com/2015/01/human-cases-of-avian-influenza.html. Accessed 29 January 2015

^c^ http://www.oie.int/en/animal-health-in-the-world/update-on-avian-influenza/. Accessed 29 January 2015

## Strategy and Methods

### Formation of a national steering committee and creation of a national vaccine deployment plan

The National Emerging Infectious Disease Coordinating Office (NEIDCO), responsible to the Prime Minister’s Office, Government of Lao PDR (GOL) organized the creation of a steering committee comprised of medical staff from hospitals, medical schools, and public health departments, along with international partners that included WHO, United Nations Children’s Fund (UNICEF), and United States Centers for Disease Control and Prevention (US CDC) The Steering Committee was tasked with developing a National Vaccine Deployment Plan, that was completed in September 2009. This plan provided guidelines and roadmap to rapid and safe deployment of A(H1N1)pdm09 vaccine, with a target coverage of > 10% of the Lao population (estimated at 6.5 million), in accordance with the WHO’s Strategic Advisory Group on Immunizations (SAGE) recommendations [6]. Targeted populations included: all government employed health care workers (HCWs) including from Ministry of Defense and Police Hospitals; pregnant women and persons with chronic illnesses aged >6 months.

The WHO PIVDI employed a phased approach to delivery of vaccine. Lao PDR received confirmation in November 2009 that they would receive 600,600 doses of donated pandemic A(H1N1)pdm09 vaccine (Panvax, Sanofi Pasteur), which arrived on February 26, 2010 [7]. Another 400,000 doses were requested to cover an additional 6.7% of the population; with receipt of vaccine (Panenza, Sanofi Pasteur) on June 16, 2010 [8].

During the first week(s) of the first of two phased vaccine deployments, deliveries were only to hospitals to ensure essential (and prioritized) healthcare workers were protected, followed by a campaign managed by the National Immunization Program (NIP); the NIP is nominally responsible for an Expanded Program on Immunization (EPI) delivering essential childhood vaccines, (e.g. measles, mumps and rubella (MMR), and tetanus toxoid (TT) to pregnant women. Following the initial campaign, remaining pandemic vaccine was provided to still un-vaccinated pregnant women through antenatal care services.

### Social mobilization for influenza A(H1N1)pdm09 vaccination

A vaccine awareness campaign was launched in March 2010 by The National Center for Information and Education for Health (NCIEH), with a ceremony in Vientiane Capital attended by ranking members of the Government in raising public understanding and support of this initiative. Additional launches were also held in each province along with local promotion activities.

Orientation training for journalists in all provinces was carried out to ensure evidence-driven information was disseminated and scare rumors could be managed through responsible reporting. The MOH also posted notices in local newspapers and arranged interviews on the national media outlets, notably radio and television. Village leaders and civic organizations such as the Lao Women’s Union and the National Committee for Mother and Child were recruited to serve as vaccination advocates in mobilizing locally targeted populations.

### Staff training for influenza A(H1N1)pdm09 vaccination

Training materials were developed jointly by the MOH and International Partners in February/March 2010, prior to the initial campaign. The curriculum included the epidemiology of influenza A(H1N1)pdm09, information on the vaccine, vaccination technique, cold chain maintenance, immunization safety, recognition and management of adverse events following immunization (AEFI), and communication with communities. Training was conducted in phases to ensure NIP staff at National, Provincial and District levels received standardized instruction using a ‘train the trainer’ approach. Local leaders from village counsels, teachers and respected monks, were also provided information in advance of the campaign to encourage community participation.

### Adverse Events Following Immunization (AEFI)/Awareness/Acceptability Survey

A survey activity was conducted in conjunction with a Public Health Emergency as part of a package of pandemic response measures. There was no unique identifying information linking survey participants with aggregate findings. Therefore, the MOH approved this survey as a non-research activity. Nevertheless, written informed voluntary consent was obtained from all survey participants. The NIP provided data presented in this report and authorized sharing for the purpose of this report.

The NEIDCO chose to assess AEFIs independent of routine NIP *passive* AEFI monitoring of vaccine activities to validate pandemic vaccine safety assurances. Trainees from the Lao Field Epidemiology Training (FET) Program developed study design and collection instruments, and fielded the survey in October and November 2010: Luang Prabang Province in the northern part of the country and Savannakhet in the south.

In Luang Prabang province, four of 12 districts were randomly selected using a random number generator. Thirty-nine villages were then further randomly sampled among these districts. In Savannakhet, four of 15 districts were randomly selected, followed by 3 to 4 villages per district. A convenience sample of 20 survey participants were selected from each village by the village head or village health volunteer. Interviews were conducted using a standardized questionnaire which included information on household demographic and vaccination information; participant health status, including recent influenza diagnoses; vaccination history for pandemic H1N1 vaccine and TT; information on signs and symptoms occurring after vaccination and history of health care visits and missed work. Voluntary participation in the survey was expressed by verbal consent, and no incentive was received for participation.

An AEFI event among a pandemic vaccine recipient was defined as any of the following symptoms within 3 days (48–72 hours) of receipt of pandemic influenza vaccine: fever, headache, nausea, local reaction (swelling and pain in injection area), myalgia, paralysis, shock, abortion or seizure.

## Results

### Vaccination doses administered

During Phase 1 of the campaign, 579,723 persons were vaccinated, accounting for 10% of the population. Of the 600,600 doses of vaccine initially received, 97% were used. An additional 311,707 (78%) of the 400,000 doses delivered in Phase II of the campaign were used. A total of 891,430 targeted persons were vaccinated with A(H1N1)pdm09 vaccine by mid-2011, achieving coverage of nearly 15% of the total population of Lao PDR (Table 2).

**Table 2:** Number of distributed influenza A(H1N1) pandemic vaccine doses by target population, 2009–2010 Lao PDR.

	Total No. of distributed vaccines	Healthcare workers	Pregnant women	Persons with Chronic Illness	Other essential personnel^a^	Total No. of vaccine doses utilized	Percent of vaccine utilized
**Phase 1**	600,600	19,864	60,969	119,033	379,857	579,723	97%
**Phase 2**	400,000	905	10,829	16,537	283,436	311,707	78%
**Estimated denominator**	NA^b^	20,769	174,532	ND^c^	ND^c^	NA^b^	NA^b^

Source: Lao PDR National Immunization Program (NIP), Annual Report on NIP 2010. Date: 20 January 2011, Anonh Xeuatvongsa, Director of National Immunization Program, Ministry of Health, Vientiane, Lao Peoples Democratic Republic, email address: anonhxeuat@gmail.com; Data submitted to through WHO: http://www.who.int/influenza_vaccines_plan/resources/h1n1_deployment_report.pdf.

^a^Other Essential Personnel include essential government workers as designated by the GOL

^b^Not Applicable

^c^No Data

Target population estimates based on projections from 2005 census data allowed for vaccine coverage determination [9]. Of an estimated 20,769 HCWs in the country, vaccine was administered to 99%. The NIP approximated 174,532 pregnant women were potentially at risk during pandemic vaccine campaign, of which 60, 969 were vaccinated, during the first phase of the campaign and 10,829 during the second: providing for 41% coverage in this group. The number of persons with chronic disease conditions was very difficult to estimate and therefore no specific target was set. A total of 135,570 persons self-identified as having a chronic disease were vaccinated during both phases. The remainder of the vaccine (663, 293 doses) was administered to other essential personnel during both phases (Table 2).

### Survey findings

There were 1,204 participants in the AEFI survey: 50% (n = 599) from Luang Prabang and 50% (n = 605) from Savannakhet Provinces. The overall mean age of respondents was 38 years (range 4–82 years) and the majority were female (58%). One-hundred-twenty-two (10%) of participants were pregnant women. Nine percent of respondents reported having an underlying medical condition or chronic illness (Table 3).

**Table 3:** Demographic characteristics of participants in the adverse events following immunization (AEFI) survey who received influenza A (H1N1) pandemic vaccine and % AEFI.

		Total(N = 1204)	n with AEFI (N = 69)	(%)AEFI
**Age in years**	5–18	3	116	3
	19–24	10	122	8
	25–64	55	908	6
	65+	1	58	2
**Sex**	Male	12	507	2
	Female	57	697	8
**Ethnicity**	Lao Loum	49	800	6
	Keum Mou	19	394	5
	Hmong	1	9	11
	Other	0	1	0
**Province**	Luang Prabang	10	599	2
	Savannakhet	59	605	10
**Occupation**	Health workers	15	163	9
	Government	0	5	0
	Student	1	38	3
	Farmer	53	998	5
Underlying medical condition^a^		105	10	10
Administered with Tetanus		321	42	13
Pregnant		122	8	7

Source: Lao PDR National Immunization Program (NIP), Anonh Xeuatvongsa, Director of National Immunization Program, Ministry of Health, Vientiane, Lao Peoples Democratic Republic, email address: anonhxeuat@gmail.com

^a^Underlying medical conditions include obesity, mild high blood pressure, asthma and diabetes

**Fig 2:**
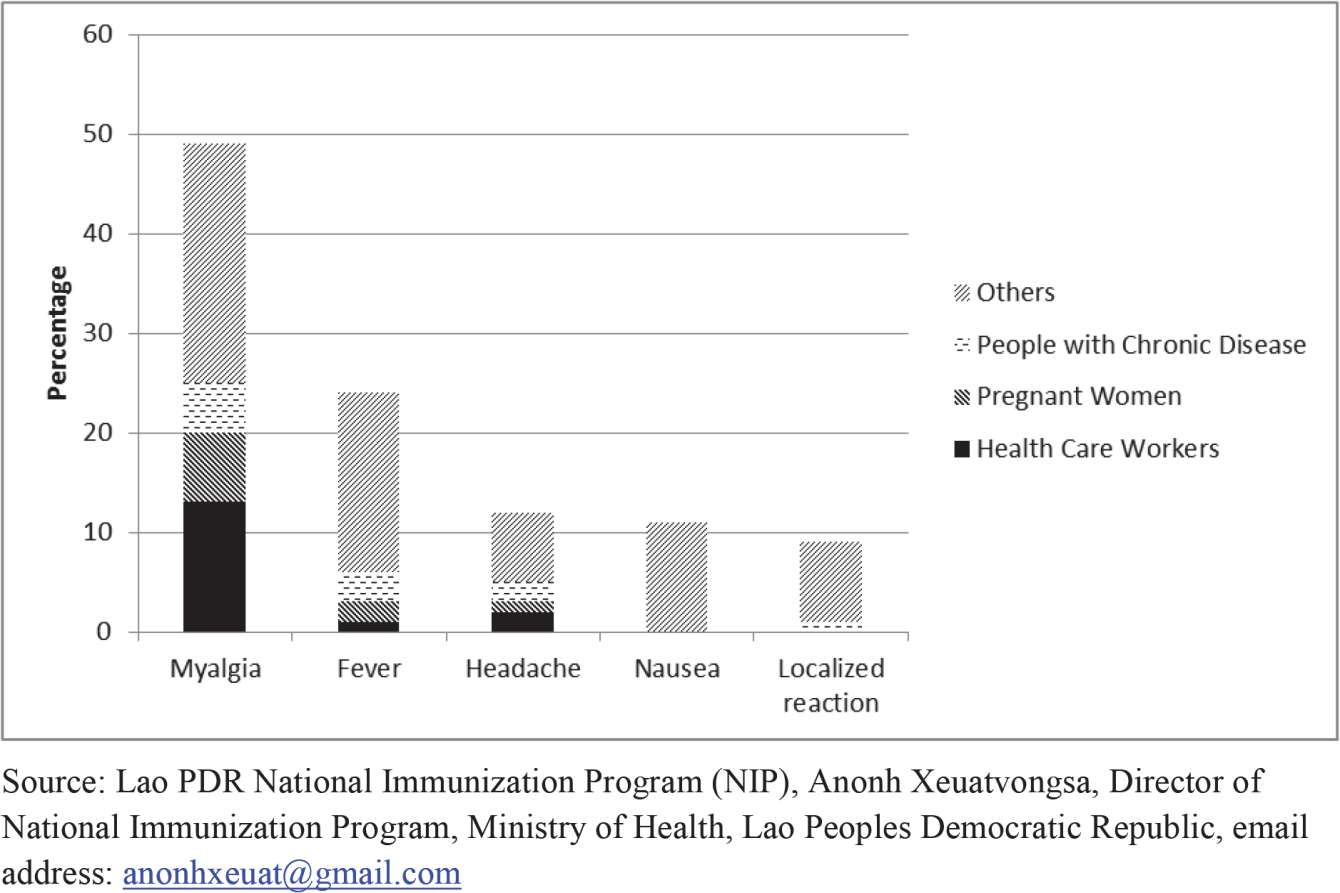
Reported AEFIs by Targeted Populations Surveyed. Lao PDR National Immunization Program (NIP), Anonh Xeuatvongsa, Director of National Immunization Program, Ministry of Health, Lao Peoples Democratic Republic, email address: anonhxeuat@gmail.com.

Among survey participants, 69 (5.7%) reported at least one mild AEFI. No serious adverse events such as seizure, paralysis or death were reported. Of AEFIs reported, most common was myalgia (72%), followed by reported fever (32%), headaches (22%) and localized reaction (7.2%) (Fig 2). The majority of participants (58%) reported only one symptom, 25% reported two symptoms and < 10% reported three or more. Overall, 122 (10%) respondents were pregnant among whom 8 (7%) reported an AEFI. No spontaneous abortions were reported. Of the 1,204 respondents, 315 (26%) reported having a maternal tetanus toxoid vaccination at the same time. Of those 42 (13%) reported an AEFI. Persons who received tetanus vaccine and influenza vaccine simultaneously were more likely to report AEFI compared to those only receiving an influenza vaccine (42/315, 13% vs. 27/889, 3%, p<0.01).

Fifteen percent of survey participants reported having never heard about pandemic influenza illness prior to the vaccination information campaign launch, whereas 55% reported learning about the pandemic influenza H1N1 vaccination campaign from a healthcare worker. Radio and television also proved to be effective modes of communication: 54% of participants reported hearing radio messages about pandemic influenza and vaccination and 42% reported learning of pandemic influenza vaccine on television. Over 98% of all respondents indicated they were satisfied with the process and would be interested in annual influenza vaccination if available.

## Discussion

The impact of A(H1N1)pdm09 as reflected from Influenza Like Illness (ILI) and Severe Acute Respiratory Illness (SARI) virological sentinel Surveillance findings was immediate and significant, as the predominate circulating influenza virus subtype beginning in July 2009 (marking the introduction of pandemic influenza in Lao PDR); 29% and 53% of recognized influenza cases were attributed to A(H1N1)pdm09 in 2009 and 2010, respectively. Prevalence estimates for Lao PDR and Mekong Region are not available because the overwhelming number of “suspected” cases forced health authorities to stop laboratory testing early on (August of 2009 in Lao PDR) because of cost considerations. Therefore the actual number of cases for 2009 and 2010 in Lao PDR (197) is based on only a small sampling and does not reflect a true prevalence measure [10].

Pandemic vaccine uptake in the Mekong Region was variable by country and targeted population(s), with coverage rates reflecting national policy, availability and public demand. In Thailand, overall usage (% of vaccine) was 77% [11]. However, survey finding showed acceptance of pandemic vaccine among Thai HCWs, as reflected in willingness to receive vaccinations, was only 60%, with those who heard about vaccine adverse events from the local broadcast media seven times more likely to reject vaccination [12]. Negative media coverage also likely contributed to low uptake among pregnant women: only 6% of the estimated 500,000 pregnant women targeted in Thailand were vaccinated [13]. Unfortunately, documentation from the other Mekong countries that carried out pandemic influenza vaccinations (Cambodia and Myanmar) reflecting uptake and acceptability in prioritized populations is notably absent from the literature.

The Lao MOH prioritized obtaining WHO-managed vaccine donations, but only after a thorough vetting of both manufacturer and independent sourced documentation attesting to vaccine safety, and with WHO endorsement and assurances, but in the absence of WHO pre-qualification status. Nevertheless, delays in fielding of vaccine some 10 months after first case recognition can be attributed to the following factors: 1) initial sensitivities arising from WHO policy requiring a signed (by the Minister of Health) liability waiver before release of vaccine stocks; 2) ministerial concerns regarding vaccine efficacy following inquiries by the Program for Appropriate Technology in Health (PATH) Organization to conduct an immunological study targeting pregnant women in conjunction with the proposed vaccination campaign being considered at the time; 3) media reports from neighboring Thailand attributing spontaneous abortions and fetal deaths to pandemic vaccine; and 4) delayed access to the vaccine (in part due to formidable logistical challenges experienced by WHO in making deliveries). Late entrance of the vaccine also created doubt within the MOH as to the value of vaccinating following the “first wave” of the pandemic. Postponed deployment attributed to late vaccine arrival indeed characterized the Mekong regional experience: 3 May 2010 in Cambodia, 4 April 2010 in Myanmar, and (late) January 2010 in Thailand [14, 15]. There was little documentation reported in the literature from Cambodia and Myanmar.

The success of pandemic influenza vaccine deployment (89% of vaccine reaching targeted populations, with 14% coverage of the total population) in Lao PDR can be largely attributed to NIP oversight and management, taking advantage of existing EPI infrastructure, e.g. staffing, logistical operational experience, data record management, cold-chain resources, advocacy channels and AEFI monitoring. The use of NIP ensured adequate population coverage (14%), exceeding WHO’s planned strategy for countries receiving donated vaccine in achieving coverage of at least 10% of the national population in two phases [16].

The self-reported rate of AEFI in the surveyed population was low: 6%; and the type of AEFIs reported described as mild to moderate, with the majority presenting systemic reactions such as myalgia and fever, followed by headache and local reaction. However, the extended lag time of greater than 4 weeks between administration of vaccine and the AEFI survey may have led to recall bias, leading to possible underreporting of adverse events by vaccinated persons. And yet NIP *passive* AEFI Surveillance yielded only fourteen AEFIs:— 13 mild and one moderate, for a rate of 1.57 AEFIs per 100,000 vaccinated population, and 1.39 AEFIs per 100,000 pregnant women vaccinated: as compared with 5,730.89 AEFIs per 100,000 vaccinated and 655.37 per 100,000 pregnant women, from the *active* AEFI Survey findings. This difference attests to the critical importance of *active* Survey Surveillance in capturing AEFI’s associated with pandemic vaccinations. The importance of a vigorous AEFI Surveillance activity when introducing pandemic (or any new) vaccine cannot be over-emphasized [11].

Findings also show that nearly half the surveyed population had heard of the pandemic vaccine’s availability either from a HCW or radio or television, reflecting a high return on the MOH’s investment in promoting public awareness.

## Conclusions

Notably, the success of the Lao pandemic vaccine initiative as demonstrated by public demand and acceptance, prompted the Ministry of Health to introduce annual seasonal influenza vaccinations supported through public-private partnerships, beginning in 2012 with deployment of 375,000 doses, followed by 100,000 and 675,000 doses in 2013 and 2014 respectively [17]. Lao PDR successfully integrated an adult pandemic and seasonal influenza vaccine activity onto a platform traditionally used to provide children’s vaccines. This allowed for an efficient use of existing infrastructure to effectively deliver vaccine to newly targeted populations.

Lastly, future vaccine approaches in countering pandemic influenza threats will be seriously flawed given the lag between pandemic recognition to vaccination, as experienced in Mekong the region as a whole in 2009–10: “It is an indefensible fact that these vaccines started to flow to poorer countries well after they began going to the countries with advance purchase arrangements” [18]. Only through local/regional pandemic vaccine manufacturing, sustained by annual seasonal influenza vaccine production and yearly deployment during inter-pandemic periods, will timely supplies be assured [13, 18].

The Lao experience in deploying pandemic influenza vaccine in 2010 serves as an example for a region faced with multiple influenza pandemic threats in the present days.
